# Integrated analysis of transcriptome and microRNAs associated with exogenous calcium-mediated enhancement of hypoxic tolerance in cucumber seedlings (*Cucumis sativus* L.)

**DOI:** 10.3389/fpls.2022.994268

**Published:** 2023-01-04

**Authors:** Lizhong He, Jun Yan, Xiaotao Ding, Haijun Jin, Hongmei Zhang, Jiawei Cui, Qiang Zhou, Jizhu Yu

**Affiliations:** ^1^ Shanghai Key Lab of Protected Horticultural Technology, Horticultural Research Institute, Shanghai Academy of Agricultural Sciences, Shanghai, China; ^2^ Shanghai Dushi Green Engineering Co., Ltd., Shanghai, China

**Keywords:** cucumber, hypoxia, microRNA, exogenous calcium, transcriptome

## Abstract

Plants often suffer from hypoxic stress due to flooding caused by extreme weather. Hypoxia usually leads to restricted oxygen supply and alters metabolic patterns from aerobic to anaerobic. Cucumber roots are fragile and highly sensitive to damage from hypoxic stress. The purpose of this study was to investigate the regulatory mechanism of exogenous calcium alleviating hypoxic stress in cucumber through transcriptome and small RNAs analysis. Three treatments were performed in this paper, including untreated-control (CK), hypoxic stress (H), and hypoxic stress + exogenous calcium treatment (H + Ca^2+^). A large number of differentially expressed genes (DEGs) were identified, 1,463 DEGs between CK vs H, 3,399 DEGs between H vs H + Ca^2+^, and 5,072 DEGs between CK vs H + Ca^2+^, respectively. KEGG analysis of DEGs showed that exogenous calcium could activate hormone signaling pathways (ethylene, ABA, IAA and cytokinin), transcription factors (MYB, MYB-related, bHLH, bZIP, and WRKY), calcium signaling and glycolysis pathway to mitigating hypoxic stress in cucumber seedlings. Additionally, miRNA and their target genes were detected and predicted between treatments. The target genes of these miRNAs revealed that auxin, cellulose synthase, and mitochondrial ribosomal related genes (Csa2G315390, Csa6G141390, Csa4G053280, and Csa6G310480) probably play in the improvement of the hypoxic tolerance of cucumber seedlings through exogenous calcium application. In short, our data adds new information to the mechanism of exogenous calcium mitigation of hypoxic stress injury in cucumber seedlings at transcriptional and post-transcriptional levels.

## 1 Introduction

In natural or artificial environments, plants are often suffering from hypoxic stress, such as flooding due to heavy rainfall and low dissolved oxygen in hydroponic culturing. The main impact of overirrigation and heavy rain is a reduction in the oxygen (O_2_) supply, as a result, the root systems are often subjected to hypoxic stress. This is because of the significantly lower diffusion capacity of oxygen in water than in atmosphere (Kaur et al., 2021). Plants have evolved a range of defence mechanisms to adapt to anoxic conditions (Ambros et al., 2022). These response to hypoxic stress include a numbers of physiological, metabolic and morphological changes, such as the formation of adventitious roots (ARs), the production of reactive oxygen species (ROS) and toxic end-products from glycolytic pathway (Xu et al., 2018). Phytohormones act as endogenous signaling also can be stimulated by hypoxic stress (Zaid et al., 2021). It had been demonstrated that ethylene (ET) and auxin act as key regulators of the aerenchyma formation in rice and cucumber under waterlogging (Fukao and Bailey-Serres, 2008; Qi et al., 2019). The oxygen deficit caused by flooding can affect the concertration of abscisic acid (ABA) and gibberellic acid (GA) in soybean seeds (Zhou et al., 2021). Besides, anoxic condition also can induce the stomatal closure and reduces the photosynthetic rate of plants. Plant growth and photosynthesis are injured by low oxygen stress, which will eventually affect the plant production (Shabala et al., 2014).

*Cucumis sativus* L. (cucumber) is an important vegetable mainly cultivated in greenhouses and other protected facilities. Under hypoxic conditions, the root system of cucumber can easily decay because of the cucumber’s strict O_2_ requirement and sensitiviness to hypoxia (Qi et al., 2019). To increase the plant adaptaion to hypoxic stress, breeding of new varieties or application of exogenous substances is an economically and environmentally way to improve plants hypoxic tolerance (Ahmed et al., 2013). Calcium (such as cytosolic Ca^2+^) plays a central role in plant signal transduction, and also acts as a signaling ion to regulate several cellular growth and development processes (Qiu et al., 2020). Various abiotic stresses can increase the concentration of Ca^2+^ in plants, including cold, drought, light, hypoxia and plant hormone (Kong et al., 2020; Li et al., 2022). For example, exogenous calcium can improve the plant growth (height, stem diameter and leaf thickness), increase the proline and soluble sugar contents of cherry tomato under waterlogging conditions (Liu et al., 2023). The foliar spray with CaCl_2_ improved the uptake of Ca^2+^ and antioxidants systems in *Thymus vulgaris* under salt stress (Zrig et al., 2021). In addition, CaCl_2_ treatment also could effectively regulated the activity of antioxidative enzymes and membrane lipid composition green peppers (*Capsicum annuum* L.) under cold stress (Zhang et al., 2021). Our previous research had found that exogenous calcium enhanced the hypoxic resistance of cucumber seedlings by mediating the photosynthesis system, respiratory metabolism enzymes, and ion transport (He et al., 2015; He et al., 2018).

In recent years, the mechanisms of corolla senescence regulatory in petunia (Wang et al., 2018a), disease resistance and fruit spine development of cucumber (Wang et al., 2018b; Liu et al., 2018) had been elucidated by high-throughput gene expression analysis of messenger RNA (mRNA) sequencing (RNA-Seq). The involvement of calcium signaling proteins in plant transcriptomic response also has been studied in *Brassica oleracea* (Tortosa et al., 2019). Metabolomic and transcriptomic analyses of cherry fruit exogenous calcium activated gene expression, most strongly those involved in plant-pathogen interaction, plant hormone signaling and MAPK signaling pathway (Michailidis et al., 2022). Exogenous calcium also can promote soluble sugars and anthocyanin accumulation in grapes skin by regulating transcription factors, including MYB, bHLH, NAC and bZIP (Yu et al., 2020). MiRNAs, as a group of small non-coding single-stranded RNA molecules, exert their regulatory effects by binding to complementary sequences in miRNAs to promote degradation or inhibiting the protein translation, leading to the suppression of the corresponding genes (Zhang et al., 2019). Increasing evidence demonstrate that miRNAs play important functions in plants’ abiotic stress response and defense processes by directly or indirectly regulating the expression of related resistance genes (Ma et al., 2014). Previous research found that miR858 plays a negative role in tomato anthocyanin biosynthesis, and inhibiting of the expression of *MIR858* results in the accumulation of anthocyanin (Chen et al., 2018). Han et al. (2016) had identified several miRNAs that may be involved in powdery mildew resistance in Chinese wild *Vitis pseudoreticulata*. In cucumber, 8 common differentially expressed miRNAs were observed that responded to the high-temperature and exogenous spermidine (Spd) (Wang et al., 2018c), 7 novel miRNAs had been predicted that may be related to the response to *Corynespora. cassiicola* (Wang et al., 2018b).

In this research, we investigated the changes in cucumber transcriptome and miRNA under hypoxia and exogenous calcium treatment by high-throughput sequencing technology. The differentially expressed genes (DEGs) and the miRNAs that responsed to hypoxic stress and exogenous calcium in cucumber were identified, which may provide useful information and theoretical support for the further study of molecular mechanisms of cucumber hypoxic tolerance and contribute to high-quality and yield cultivation of cucumber production.

## 2 Materials and methods

### 2.1 Plant materials

Cucumber seeds (*Cucumis sativus* L. cv. Jinchun No. 2, sensitive to hypoxia) were washed with deionized water and then sown in two plastic trays (41 cm × 41 cm × 5 cm) containing quartz sand. The germinated cucumber seedlings were grown in a greenhouse in Shanghai (31.4°N, 121.5° E, P. R. China). The day and night temperatures of the greenhouse were maintained at 25 ± 2°C (day) and 17 ± 2°C (night), relative humidity (RH) from 75 to 90%, and maximum photosynthesis photon flux density (PPFD) of natural light about 1200 μmol m^-2^ s^-1^. After the cotyledons of seedlings had expanded, the half-strength Hoagland nutrient solution was used to irrigate the cucumber seedlings regularly. When plants had 2 fully developed leaves, cucumber seedlings were transferred into every three plastic containers (50 cm × 32 cm × 18 cm), and the containers were filled with full-strength Hoagland nutrient solution. The nutrient solutions were maintained at 20-25°C and using an air pump to keep the dissolved oxygen at 8.0 ± 0.2 mg L^-1^. After the third leaves were fully developed, the cucumber seedlings were treated as follows: 1) Control (CK): cucumber seedlings were cultured with full-strength Hoagland solution (containing 2 mM Ca^2+^) and the dissolved oxygen (DO) level was kept at 8.0 ± 0.2 mg L^-1^; 2) Hypoxic treatment (H): cucumber seedlings were cultured with full-strength Hoagland solution (containing 2 mM Ca^2+^) and the N_2_ gas was pumped to maintain the DO level at 1.0 ± 0.1 mg L^-1^; 3) Hypoxia + CaCl_2_ treatment (H + Ca^2+^): full-strength Hoagland solution was added with 4 mM CaCl_2_, the DO level and its control method were same as the hypoxic treatment.

Following 3d of treatment, the roots of plants under three treatments were harvested, immediately frozen in liquid nitrogen, and stored in a -80°C refrigerator for further transcriptome analysis. This experiment uses a random block design and three biological replicates for each analytical experiment of the transcriptome analyses and the quantitative real-time PCR (qRT- PCR) experiments were harvested.

### 2.2 High-throughput sequencing of transcriptome and small RNAs in cucumber roots

The equal amounts of cucumber roots from three treatments in three independent biological replicates were pooled and used to construct a cucumber root transcriptome library. Total RNA was isolated using a plant RNA Kit (Trizol reagent, Tiangen Technology, Co., Ltd., Beijing, China) according to the manufacturer’s protocol and the concentration of RNA was measured and followed with previously reported (Nie et al., 2015). The sRNAs (sized at 18-30nt) were separated and fragmented, and then the isolated sRNAs were added to 5’- and 3’-RNA adaptors, reverse transcription, purification, size selection, and used for High-throughput sequencing last. Both transcriptome and small RNAs were performed by using HiSeq™ 4000 sequencing system (Illumina, Inc., San Diego, CA, USA).

### 2.3 RNA-Seq read processing, assembly, and transcriptome sequence analysis

The raw reads were acquired from original sequencing data and the clean reads were obtained after removing low-quality (< Q20) reads, mismatches, adaptor sequences, and reads containing polyA, ployN, and polyT ends. These clean reads were then matched to the cucumber reference sequences through the software Soap2 (Li et al., 2009) and Tophat2 (Kim et al., 2013) in http://cucurbitgenomics.org. The downstream analyses were based on the alignment results of clean data.

To control the false discovery rate (FDR), differentially expressed gene (DEGs) analysis of three comparisons was performed with an adjusted P-value < 0.05 using the approach according to Benjamini et al. (2001). The |log2 fold changes (FC)| ≥ 1 and FDR ≤ 0.05 were used as filter criteria to screen the significant DEG (Audic and Claverie, 1997). Gene Ontology (GO) and Kyoto Encyclopedia of Genes and Genomes (KEGG) databases were used to explore the biological function and signaling pathways of the identified significant DEGs. For GO annotation, the DEGs were searched in the NCBI database with the E < 10^-5^ using software BLAST (blastall 2.2.260). KEGG pathways were identified by a BLAST search of the KEGG database based on p-values and adjusted q-values pathways (Young et al., 2010).

We also used Mapman software (v3.5.1R2) to visualize and identify some gene families that may play key roles in the regulation of hypoxic metabolism of cucumber seedlings, such as hormone biosynthesis and signaling pathways, transcription factors, calcium signaling, and glycolysis pathway (Usadel et al., 2005). Genes were classified into a set of hierarchical functional categories (BINs), based on Mapman BIN from the ITAG2.4 annotation (Wang et al., 2018a). The level of significance was set at FDR < 0.05 (Fukushima et al., 2018).

### 2.4 Isolation, high-throughput sequencing and bioinformatic analysis of miRNA

After filtering the low-quality and contaminated sequence (adaptor, polyA, reads shorter than 18nt, readers with 5’primer or without 3’primer, readers without the insert), RNA samples were used to generate three sRNA libraries and for the further advanced analysis. Then clean reads were BLASTed against the NCBI GeneBank, Rfam (Rfam 11.0, RNA family), and MiRbase 21.0 (http://www.mirbase.org/index.shtml). The matched non-coding miRNAs including ribosomal RNAs (rRNAs), transfer RNAs (tRNAs), small nucleolar RNAs (snoRNAs), and small nuclear RNAs (snRNAs) were found and removed as more as possible. To identify known miRNAs, the specific strategies were as follows: 1) the miRNA precursor sequence must be completely compared to the MIRbase (no mismatch); 2) the comparison of mature miRNA in the MIRbase database allowed mismatch, but there was at least 16nt coverage, the coverage part without mismatch. For sequences that were not annotated to any information, we used Mireap software (http://sourceforge.net/projects/mireap/) to predict novel miRNAs. Differentially expressed miRNAs were selected according to the difference in expression |fold change=log_2_-ratio| > 1. miRNAs with fold changes (log2) less than 1or greater than 1, and *p* < 0.05 were considered to have significantly altered expression.

### 2.5 Prediction and annotation of target genes for miRNAs

The online software plant small RNA target server (psRNATarget; http://plant-grn.noble.org/psRNATarget/) was used to predict the potential target genes of the corresponding identified miRNAs (Dai and Zhao, 2011). GO enrichment and KEGG pathway analysis were performed through Blast2GO program to investigate the potential functions of these annotated miRNAs and their targets.

### 2.6 Real-time quantitative RT-PCR

Quantification was performed with a two-step reaction process: reverse transcription (RT) and PCR. Each RT reaction consisted of 0.5μg RNA, 2μl of 5 × *TransScript* All-in-one SuperMix for qPCR and 0.5μl of gDNA Remover, in a total volume of 10μl. Reactions were performed in a GeneAmp^®^ PCR System 9700 (Applied Biosystems, USA) for 15 min at 42°C, 5 s at 85°C. The 10 μl RT reaction mix was then diluted × 10 in nuclease-free water and held at -20°C.

Real-time PCR was performed using LightCycler^®^ 480 II Real-time PCR Instrument (Roche, Swiss) with 10μl PCR reaction mixture that included 1μl of cDNA, 5μl of 2×PerfectStart™ Green qPCR SuperMix, 0.2μl of forward primer, 0.2μl of reverse primer and 3.6μl of nuclease-free water. Reactions were incubated in a 384-well optical plate (Roche, Swiss) at 94°C for 30s, followed by 45 cycles of 94°C for 5s, 60°C for 30s. Each sample was run in triplicate for analysis. At the end of the PCR cycles, melting curve analysis was performed to validate the specific generation of the expected PCR product. The primer sequences were designed in the laboratory and synthesized by TsingKe Biotech based on the mRNA sequences obtained from the NCBI database, and were showed in **Table 1**.

**Table 1:** Primers used for quantitative real-time PCR (qRT-PCR) analysis.

Gene id	Gene name	Forward primer (5′–3′)	Reverse primer (3′–5′)
csa7g448680	IAA	CACAGTCTATGTTGGTAAGTCAC	ACGACTTATCAACCAACTCT
csa4g618520	ABA	ATCAAGCTTCCCAATGACA	AAGCGTATAGATTTACCCTCG
csa7g375820	Ethylene	GTGGAACAATGATCACAATCAG	ACATCGTCTCTTCGGC
csa2g023880	LOX 1	CTTGACCCAAATGTTTATGGGA	ACTCACCTCATCAACCGTAA
csa1g573650	bZIP	GAAACTGAACCGGAGCG	CTTGATTTCTACGGCGTTTG
csa3g727990	WRKY	CTTTGATCGAAGAAAAGCAGC	GATTCTGGTGAGTAGATCTTGG

### 2.7 Statistical analysis

The data were analyzed with the SAS (version 9.2, SAS Institute, Cary, NC, USA) using Duncan’s multiple to identify differences between means (p < 0.05).

## 3 Result

### 3.1 Analysis of DEGs under different treatments

As shown in **Table 2**, RNA sequencing from three treatments (CK, H and H + Ca^2+^) generated 54,030,338, 54,029,014 and 54,028,994 raw reads, and 47,674,226, 46,884,408 and 47,108,756 clean reads, respectively. These clean reads were then mapped to the cucumber reference genome, resulting in the generation of 88.7% (CK), 87.8% (H), and 89.3% (H + Ca^2+^) matched reads in three treatments. A few more mapped reads from H + Ca^2+^ treatment indicated that some specific genes may be different expressed with exogenous calcium treatment under hypoxic stress. Further analysis revealed that 26,894,756 reads (56.41%) in CK library, 26,422,549 reads (56.40%) in H library and 25,758,445 reads (54.68%) in H + Ca^2+^ library were uniquely matched (**Table 2**).

**Table 2:** Summary of DGE sequencing and mapped reads.

Type	CK			H			H + Ca^2+^		
	Count	Q20 (%)	Percent (%)	Count	Q20 (%)	Percent (%)	Count	Q20 (%)	Percent (%)
Raw reads	54,030,338	95.25		54,029,014	95.07		54,028,994	95.02	
Clean reads	47,674,226	97.14		46,884,408	97.17		47,108,756	97.15	
Total mapped			88.7			87.8			89.3
<=5bp mismatch	5,812,243		12.19	5,697,747		12.16	5,671,732		12.04
unique match	26,894,756		56.41	26,422,549		56.40	25,758,445		54.68
multi-position match	353,190		0.74	315,396		0.67	37,4223		0.79

### 3.2 Identification and functional enrichment analysis of DEGs

**Figure 1:**
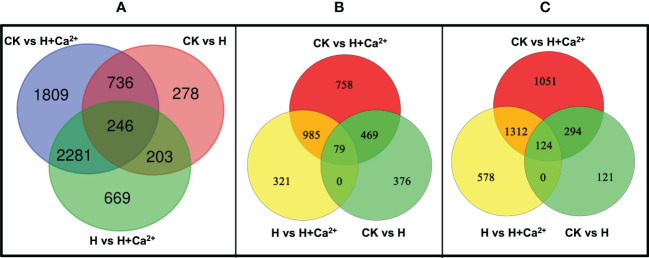
Venn diagram of the differentially expressed genes (DEGs) in pairwise analysis. **(A)** total DEGs, **(B)** upregulated DEGs, **(C)** downregulated DEGs.

FPKM method (|log2Ratio| ≥ 1 and FDR ≤ 0.005) were used to analyze the transcript abundance of genes from three libraries. Differential expression analysis was conducted by pairwise comparisons, a total of 1,463, 5,072, and 3,399 DEGs were identified between CK vs H, CK vs H + Ca^2+^, and H vs H + Ca^2+^, respectively (**Figure 1A**). Except for CK vs H, there are more downregulated DEGs in both H vs H+ Ca^2+^ and CK vs H+ Ca^2+^ than upregulated DEGs (**Figures 1B, C**). In CK vs H, 63.16% genes (924) were upregulated and 36.84% genes (539) were downregulated; 45.17% genes were upregulated and 54.83% genes were downregulated in CK vs H + Ca^2+^; 40.75% genes were upregulated and 59.25% genes were downregulated in H vs H + Ca^2+^. Compared with CK, hypoxic stress resulted in 924 genes upregulated and 539 genes downregulated (CK vs H). After applying exogenous calcium (H vs H + Ca^2+^), the number of upregulated genes increased 49.89% to 1385 and the downregulated genes increased 273.65% to 2014 (**Figures 1B, C**).

**Figure 2:**
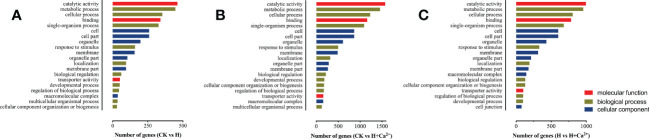
Top 20 significantly enriched GO terms of the differentially expressed genes (DEGs). **(A)** CK vs H (control treatment compared with hypoxic treatment), **(B)**: CK vs H + Ca^2+^ (control treatment compared with hypoxia + CaCl_2_ treatment), **(C)**: H vs H + Ca^2+^ (hypoxic treatment compared with hypoxia + CaCl_2_ treatment) Detailed information on each gene and its expression level is listed in **Supplementary Table S1**.

To better explore the functions of these identified DEGs, we executed a Gene Ontology (GO) assignment to classify transcriptomic regulation induced by hypoxia and exogenous calcium. These DEGs were annotated in three main GO categories: “biological process (BP)”, “cellular component (CC)”, and “molecular function (MF)”. The top 20 GO enrichment terms were almost the same between the three comparisons, except for the number of genes (**Figures 2A-C** and **Supplementary table S1**). The terms “catalytic activity” (GO: 0003824) and “binding” (GO: 0005488) were the major groups in the molecular function category; “metabolic process” (GO: 0008152), “cellular process” (GO: 0009987) and “single-organism process” (GO: 0044699) were the dominant groups in the biological process. For the cellular component, numbers of genes were highly enriched in “cell” (GO: 005623), “cell part” (GO: 0044464), “organelle” (GO: 0043226), and “membrane” (GO: 0016020) (**Figure 2A–C**).

Under hypoxic stress (CK vs H), there are 66.4% DEGs (2,508 of 3,777) upregulated, and the ontology of BP, CC, and MF had 1,703 (45.1%), 1,168 (30.9%) and 906 (24.0%) DEGs, respectively (**Figure 2A** and **supplementary table S1**). In the comparison of CK vs H + Ca^2+^, 62.2% DEGs were downregulated (7,863 of 12,649), and the ontology of BP, CC, and MF had 5,801 (45.9%), 3,773 (29.8%), and 3075 (24.3%) DEGs, respectively (**Figure 2B** and **supplementary table S2**). In the H vs H + Ca^2+^, the 69.5% DEGs were also downregulated (5,813 of 8,365), and the ontology of BP, CC, and MF had 3,701 (44.2%), 2,655 (31.7%) and 2009 (24.0%) DEGs, respectively (**Figure 2C** and **supplementary table S3**).

**Figure 3:**
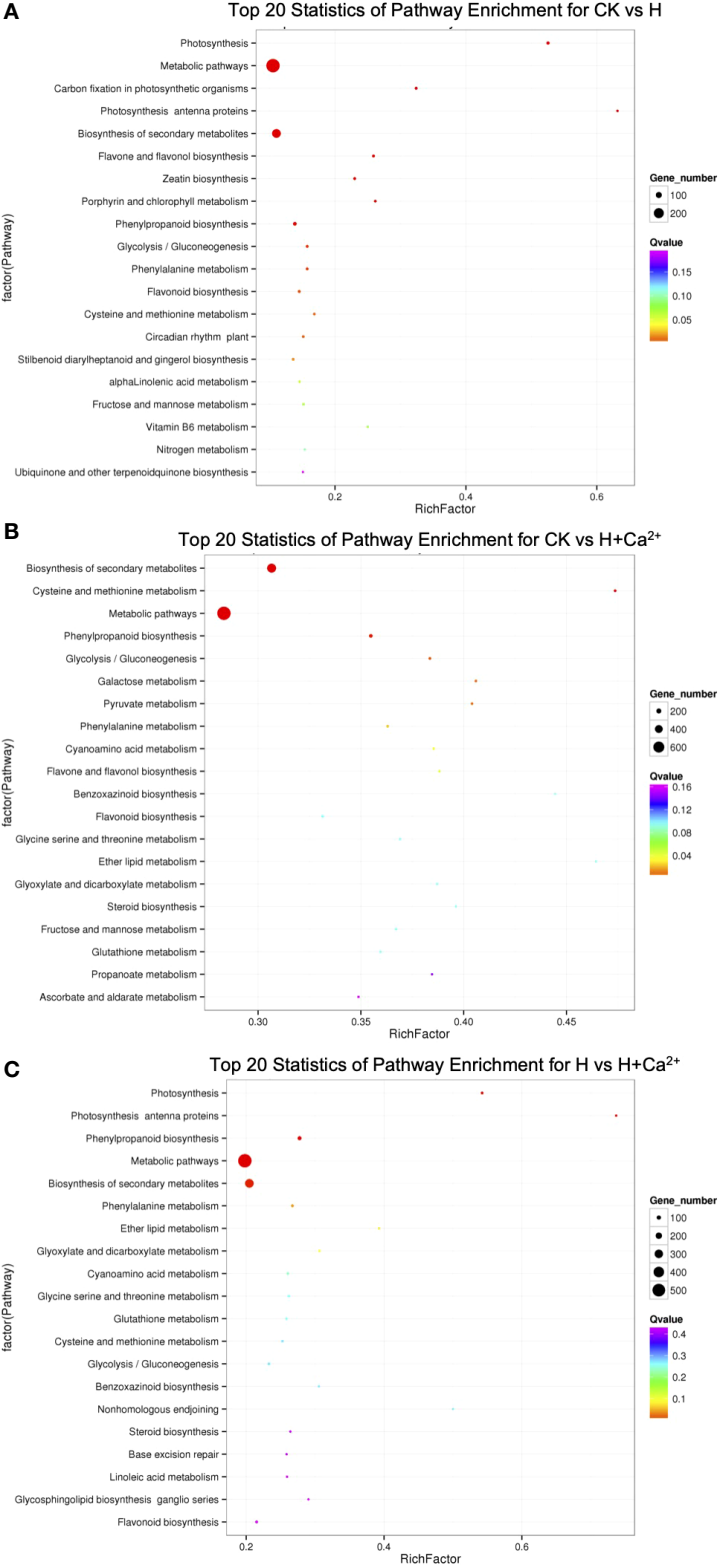
KEGG pathway enrichment analysis. The x-axis represented the rich factor, and the y-axis indicated the enriched KEGG pathway. **(A)** CK vs H (control treatment compared with hypoxic treatment), **(B)** CK vs H + Ca^2+^ (control treatment compared with hypoxia + CaCl_2_ treatment), **(C)** H vs H + Ca^2+^ (hypoxic treatment compared with hypoxia + CaCl_2_ treatment).

For an exploration of the putative active biological pathways of the DEGs, the identified DEGs were mapped to KEGG database with a Q-value ≤ 0.05 (**Figure 3** and Supplied **Table S2**). The dominant pathway was “metabolic pathways” (ko01100) in three comparisons (284 of 834 DEGs in CK vs H, 764 of 2901 DEGs in CK vs H+Ca^2+^, and 534 of 1981 DEGs in H vs H+Ca^2+^). The second enrichment pathway was “biosynthesis of secondary metabolism” (ko01110), it had 168, 466, and 311 DEGs in CK vs H, CK vs H+Ca^2+^, and H vs H+Ca^2+^, respectively. Moreover, under exogenous calcium (CK vs H+Ca^2+^) treatment, we found some pathways were significantly enriched, including “phenylpropanoid biosynthesis” (ko00940, 110 DEGs), “glycolysis/gluconeogenesis” (ko00010, 56 DEGs), “galactose metabolism” (ko00052, 41 DEGs) and “pyruvate metabolism” (ko00620, 40 DEGs).

### 3.3 The expression of DEGs in hormone biosynthesis and signaling pathways

To understand the regulatory role of crucial DEGs in cucumber hypoxic resistance with exogenous calcium, all DEGs related to hormone signaling, transcription factors, calcium signaling, and glycolysis across these three comparisons were analyzed with Mapman software.

**Figure 4:**
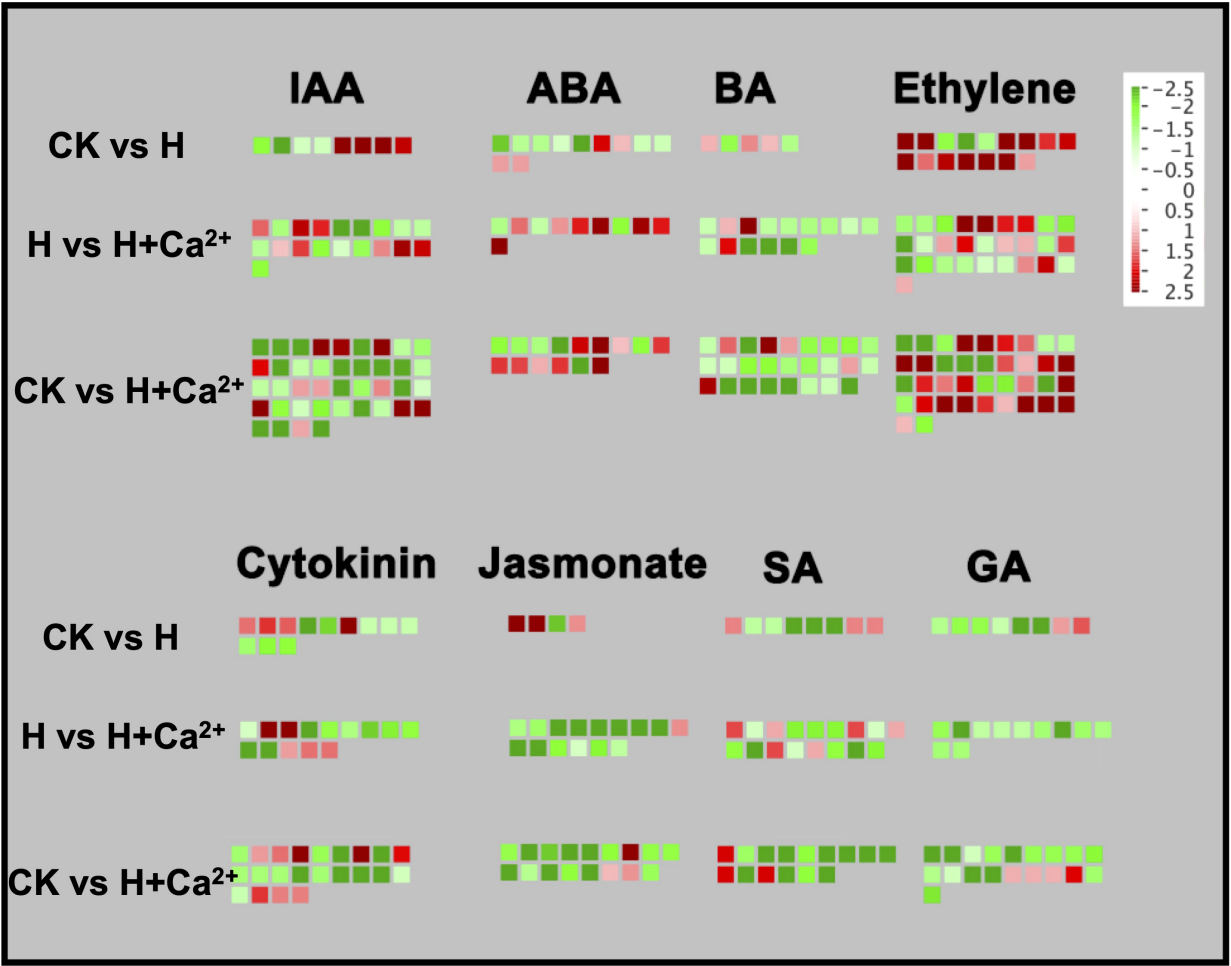
Display of gene expression involved in hormone biosynthesis and signaling pathway. Significantly DEGs (log2 fold changes (FC) ≥1, FDR ≤0.05) were visualized using Mapman software and organized into functional categories (BINs). Green indicates a decrease and red an increase in gene expression (see color set scale on top right corner). Detailed information on each gene and its expression level is listed in **Supplementary Table S3**.

In the hormonal signaling pathway, the largest numbers of identified DEGs were related to the auxin and ethylene signaling pathways, followed by DEGs in the cytokinin, BA (6-benzylaminopurine signaling), SA (salicylic signaling), GA (gibberellin signaling), ABA (abscisic acid signaling) and jasmonate pathway (**Figure 4**). Predominantly of DEGs involved in ethylene biosynthesis and signaling pathways were upregulated through the CK vs H (81.25%, 13 out of 16), but most DEGs were downregulated (57.14%, 16 out of 28) after exogenous calcium was applied (H vs H+Ca^2+^). The DEGs belonged to response to stress (csa4g003620), ethylene response factors (ERFs) (csa5g167110 and csa7g375820), and ACC oxidase (csa2g000520 and csa6g511860) were found upregulated under hypoxia and downregulated under exogenous calcium treatment. Exogenous calcium treatment also increased some ERFs, such as csa2g177210, csa3g017320, csa3g135120, csa4g641590, csa2g361860 (DNA binding transcription factor) and csa1g004080 (ATP-dependent helicase) (**Figure 4** and **Supplementary table S3**). For the auxin pathway, we identified four genes that belonged to the auxin-responsive protein family, one gene (csa7g448680) was found to be upregulated under hypoxic stress (CK vs H) and downregulated after exogenous calcium was applied (H vs H+Ca^2+^), the other three DEGs (csa6g092560, csa6g446320, and csa6g452710) were found upregulated under exogenous calcium treatment compared to hypoxic treatment. For the cytokinin pathway, four DEGs (csa1g589060, csa1g589070, csa2g362450, and csa5g175820) related to cytokinin oxidase (*CKX*) were upregulated under hypoxic stress (CK vs H) and the csa5g175820 were downregulated in H vs H+Ca^2+^. The exogenous calcium treatment also upregulated four DEGs involved in histidine kinases (csa6g095330 and csa6g110860) and UDP-Glycosyltransferase (csa3g743980 and csa3g745000). For the ABA pathway, hypoxic stress downregulated most identified DEGs (6 out of 9), and three DEGs were upregulated in CK vs H, including *ABA 8’-hydroxylase* (csa4g056600) and *HVA22* (csa1g001410). (**Figure 4** and **Supplementary table S3**). Hypoxic stress (CK vs H) significantly upregulated Jasmonate-related DEGs, including *LOX1* (csa2g023880 and csa2g023890) and *JAZ5* (csa3g645940). When exogenous calcium was applied, the majority of identified DEGs (14 out of 15) were downregulated, and the expression of csa2g023880 and csa2g023890 were also found to be downregulated in H vs H+Ca^2+^. Predominantly DEGs involved in the SA pathway were downregulated under hypoxic stress. The csa3g635870, involved in the tryptophan synthesis pathway, was upregulated in CK vs H, but downregulated under exogenous calcium treatment (H vs H+Ca^2+^). For GA pathway, one gene (csa2g368260) that responds to gibberellin stimulus was upregulated, and all genes were downregulated when exogenous calcium was applied (H vs H+Ca^2+^). For the BA pathway, the DEGs involved in sterol biosynthesis (csa4g007710) and lipid metabolic process (csa5g612880) were found upregulated under hypoxic stress (CK vs H). After applying exogenous calcium, most DEGs were downregulated compared to stress treatment (H vs H+Ca^2+^), two DEGs involved in the leucine-rich repeat protein kinase family (csa1g011510) and lipid metabolic process (csa7g007840) were found upregulated (**Figure 4** and **Supplementary table S3**).

### 3.4 Differential expression related to transcription factors, calcium signaling and glycolysis pathway

**Figure 5:**
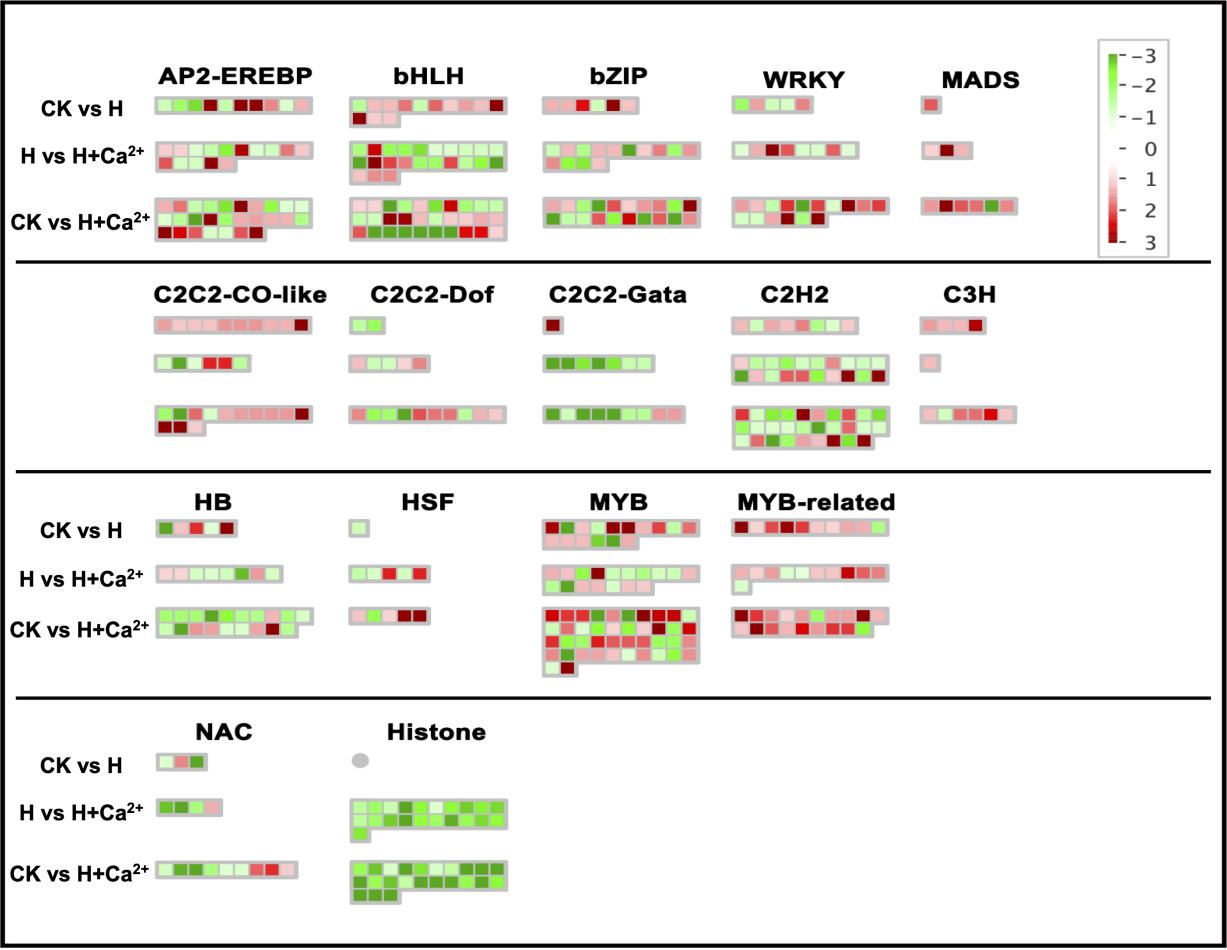
Display of gene expression of transcription factors. Significantly DEGs (log2 fold changes (FC) ≥1, FDR ≤0.05) were visualized using Mapman software and organized into functional categories (BINs). Green indicates a decrease and red an increase in gene expression (see color set scale on top right corner). Detailed information on each gene and its expression level is listed in **Supplementary Table S4**.

The different expressions of specific transcription factors (TFs) under three treatments were also analyzed with Mapman. The major TFs were identified in AP2-EREBP, bHLH, bZIP, MYB, WRKY, and C2C2-CO-like (**Figure 5** and **Supplementary table S4**). The upregulated DEGs involved in MYB, MYB-related, and C2C2-CO-like were significantly enriched under hypoxic stress. Two genes (csa2g049890 and csa2g247060) related to encoding the MYB transcription factor and one gene (csa5g610520) related to encoding B-box zinc finger family protein were upregulated in CK vs H, but downregulated in H vs H+Ca^2+^. In bHLH and bZIP, we found four DEGs (csa1g555600, csa2g263910, csa3g893390, and csa1g573650) upregulated in CK vs H and downregulated in H vs H+Ca^2+^. Two DEGs (csa2g263910 and csa4g463190) were upregulated in CK vs H and H vs H+Ca^2+^. For WRKYs, one gene (csa3g727990) related to JA-signaling was found downregulated under hypoxic stress (CK vs H) and was upregulated under exogenous calcium treatment (H vs H+Ca^2+^).

**Figure 6:**
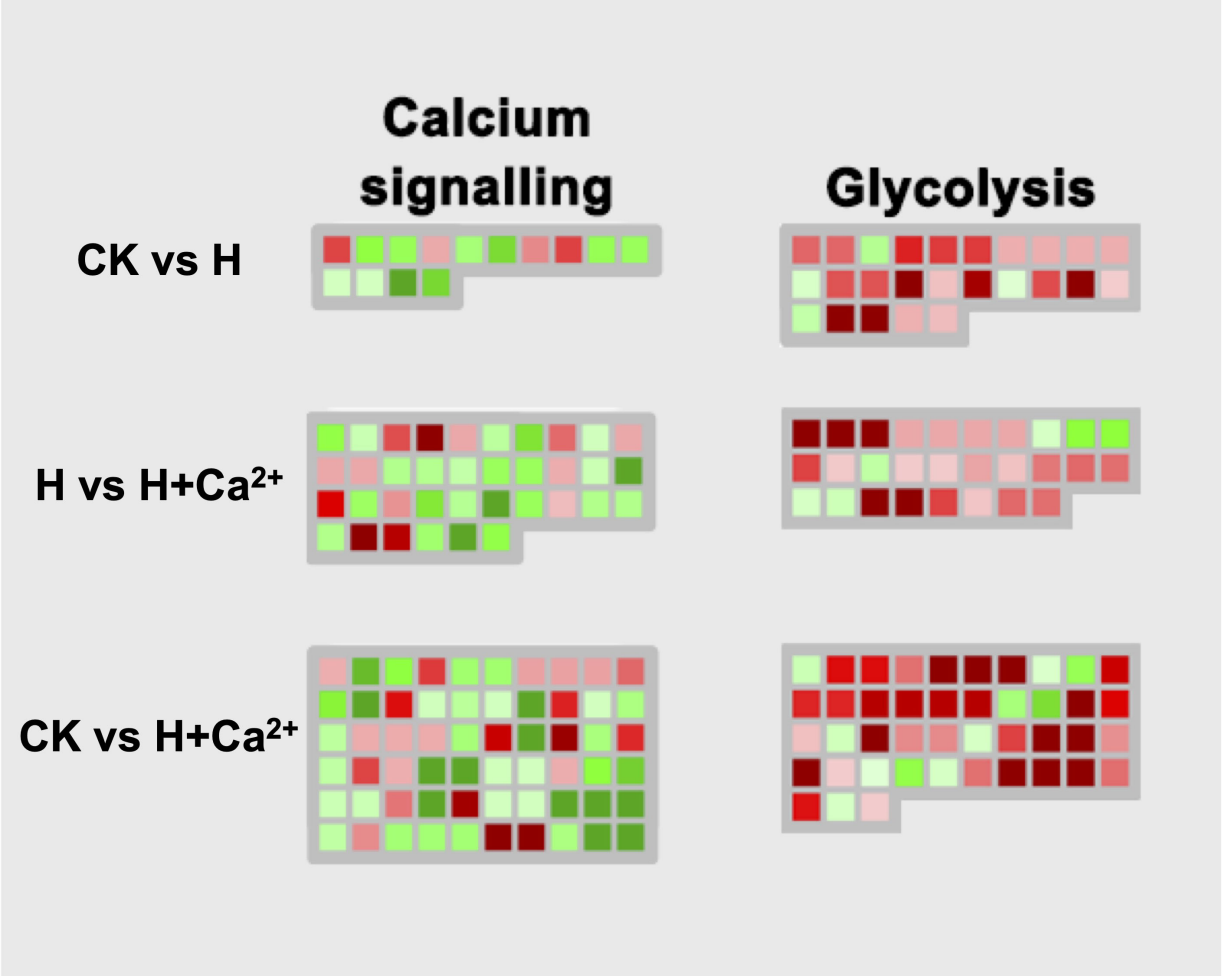
Display of gene expression of calcium signaling and glycolysis. Significantly DEGs (log2 fold changes (FC) ≥1, FDR ≤0.05) were visualized using Mapman software and organized into functional categories (BINs). Green indicates a decrease and red an increase in gene expression (see color set scale on top right corner). Detailed information on each gene and its expression level is listed in **Supplementary Table S5**.

There were a total of 13 calcium signaling-related DEGs identified, and 4 DEGs (csa2g336720, csa3g171210, csa3g912380, and csa4g091940) were upregulated under hypoxic stress (CK vs H). After applying the exogenous calcium, 31 DEGs were identified and 7 DEGs were found upregulated (H vs H+Ca^2+^). These upregulated DEGs including CDPK-related kinase1 (csa4g430830), calcium-dependent protein kinase 21 (csa6g513780) and calcium transporting ATPase (csa7g033290). For the glycolysis pathway, the majority of identified DEGs were upregulated under hypoxic stress and exogenous calcium treatment. There are four DEGs involved in encoding glyceraldehyde-3-phosphate dehydrogenase (csa1g050240), aldolase (csa3g750920), pyruvate kinase (csa5g580610), and phosphoglycerate (csa6g151120) were upregulated in CK vs H and further upregulated in H vs H + Ca^2+^ (**Figure 6** and **Supplementary table S5**).

### 3.5 Identification of differentially expressed miRNAs and their targets under different treatments

**Figure 7:**
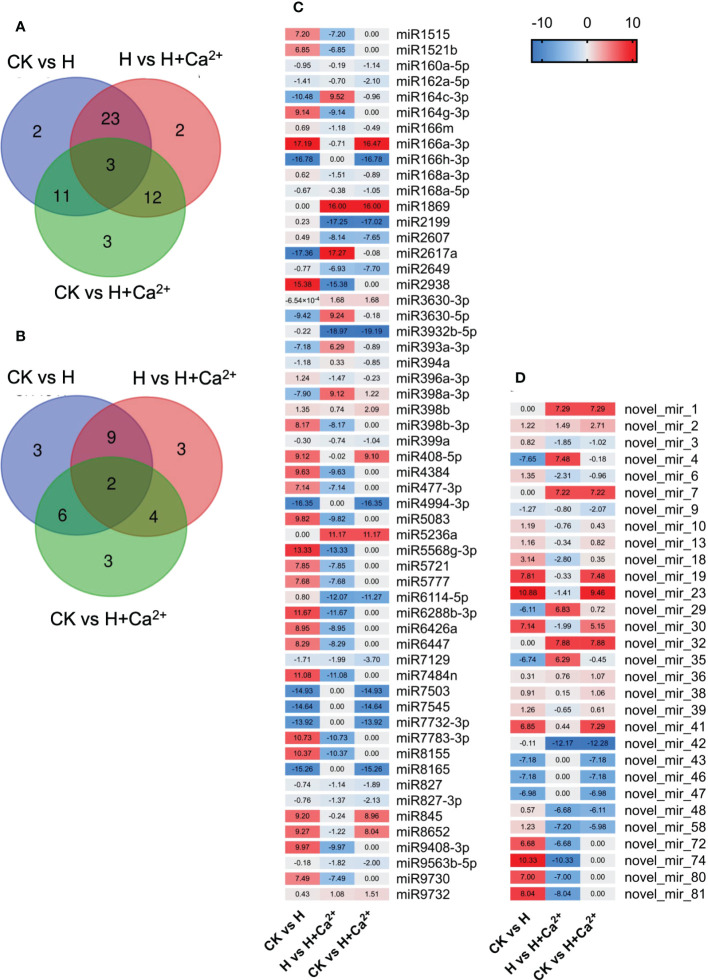
Analysis of differentially expressed miRNAs of cucumber under hypoxia and hypoxia + CaCl_2_exogenous calcium treatment. **(A)** Venn diagrams for analysis of know miRNAs differentially expressed in the CK vs H, H vs H + Ca^2+^ and CK vs H + Ca^2+^ comparison. **(B)** Venn diagrams for analysis of novel miRNAs differentially expressed in the CK vs H, H vs H + Ca^2+^ and CK vs H + Ca^2+^ comparison. **(C)** Heatmap of differentially expressed known miRNAs. **(D)** Heatmap of differentially expressed novel miRNAs. The names of miRNA are shown in brackets. Blue indicates relatively low expression; red indicates relatively high expression. The heatmap relative value were calculate by log2 fold changes (CK/H), log2 fold changes (H/H + Ca^2+^) and log2 fold changes (CK/H + Ca^2+^), respectively.

We further analyzed the miRNA expression that responded to the hypoxic stress and exogenous calcium. A total of 56 known miRNAs and 30 novel miRNAs were identified to be differentially expressed and the Venn diagram showed the numbers of common and specific differentially expressed miRNAs in three comparison pairs (**Figures 7A, B**). Under hypoxic stress, the known miRNAs, such as miR166a-3p, miR2938, miR5568g-3p, miR6288b-3p, miR7484n, and miR8155, were upregulated and the miR164c-3p, miR166h-3p, miR2617a, miR4994-3p, miR7503, miR7545, miR7732-3p, and miR8165 were downregulated (**Figure 7C**, CK vs H). When exogenous calcium was applied, the known miRNAs, such as miR164c-3p, miR1869, miR2617a, miR3630-5p, miR398a-3p, and miR5236a, were upregulated and the miR2199, miR2938, miR3932b-5p, miR5568g-3p, miR6114-5p, miR6288b-3p and miR7484n were downregulated, compared to hypoxic treatment (**Figure 7C**, H vs H+Ca^2+^). As for novel miRNAs, the novel_mir_4, novel_mir_29, and novel_mir_35 were found downregulated under hypoxic stress and upregulated after using exogenous calcium. In addition, novel_mir_72, novel_mir_74, novel_mir_80, and novel_mir_81 were upregulated under hypoxic treatment and downregulated in response to exogenous calcium (**Figure 7D** and **Supplementary table S6**).

The target genes of known and novel miRNAs were predicted to understand the biological functions of corresponding miRNAs. As shown in **Table 3**, several target genes of miR160a-5p were annotated as auxin response factors, the target genes of miR164c-3p encoded cellulose synthase-like protein, and the target genes of miR166m and miR2938 were related to a mitochondrial carrier protein and mitochondrial 28S ribosomal protein, respectively (**Table 3** and **Supplementary table S7**). Some novel miRNAs were found to be involved in ubiquitin-protein ligase (novel_mir_4), beta-galactosidase (novel_mir_58), and cellulose synthase-like protein (novel_mir_81).

**Table 3:** Candidate miRNA and their targets of know and novel miRNAs.

miRNA name	Sequence of miRNA	Target gene ID	Target gene annotation
miR160a-5p	UGCCUGGCUCCCUGUAUGCCA	Csa2G315390	auxin response factor 17
		Csa6G141390	auxin response factor 18-like
		Csa6G405890	auxin response factor 18-like
		Csa6G445210	auxin response factor 18
miR164c-3p	CAUGUGCUCUUCUUCUCCAAC	Csa4G007600	uncharacterized protein
		Csa4G053280	cellulose synthase-like protein
miR166a-3p	UCGGACCAGGCUUCAUUCCCC	Csa6G141360	homeobox-leucine zipper protein REVOLUTA isoform X2
		Csa6G525430	homeobox-leucine zipper protein ATHB-14-like
		Csa7G452940	homeobox-leucine zipper protein ATHB-15 isoform X2
		Csa1G538230	homeobox-leucine zipper protein ATHB-14-like
miR166m	UCGGACCAGGCUUCAUUCCCU	Csa3G895630	homeobox-leucine zipper protein
		Csa5G576830	mitochondrial carrier protein
miR168a-5p	UCGCUUGGUGCAGGUCGGGAA	Csa1G031900	eukaryotic translation initiation factor 2c
miR2617a	CGAGUGUGAGCAUGCCUGUU	Csa1G569300	26S protease regulatory subunit 6A homolog
miR2938	AUCUUCUGAGAAGGGUUCGAG	Csa6G310480	mitochondrial 28S ribosomal protein S29-like protein
miR394a	UUGGCAUUCUGUCCACCUCC	Csa5G184300	probable methyltransferase PMT11
		Csa6G087700	F-box only protein 6 isoform X1
miR4994-3p	UAAUUCUAGAGCUAAUACA	Csa3G733920	gamma-glutamyl phosphate reductase
miR7503	AGACCGAUAGCGAACAAGUAGA	Csa6G239680	transcript antisense to ribosomal RNA protein
miR8155	UAUCCUGGCUGCGGAACCA	Csa6G518120	homeodomain-like transcription factor superfamily protein
		Csa6G521090	60S ribosomal protein L32
novel_mir_4	CACGUGCUCCCUUUCUCCAAC	Csa6G538730	ubiquitin-protein ligase
novel_mir_10	UUCAAGAAAGCUGUGGGAGA	Csa1G032490	kelch repeat-containing F-box family protein
novel_mir_32	UCAGUAGGAACGAUACAAUCA	Csa1G002780	HAD-superfamily hydrolase
novel_mir_36	UUCAUGGGUCUCUGUUUUUAG	Csa4G003750	pectinesterase inhibitor
		Csa5G152810	mitogen-activated protein kinase 4
novel_mir_58	UAAGAGCUUCGAUAGUAACAUG	Csa1G597710	beta-galactosidase
novel_mir_81	CAUGUGCUCUUCUUCUCCAAC	Csa4G007600	putative uncharacterized protein NEF1-2
		Csa4G053280	cellulose synthase-like protein

### 3.6 Verification of RNA-seq results with qRT-PCR

**Figure 8:**
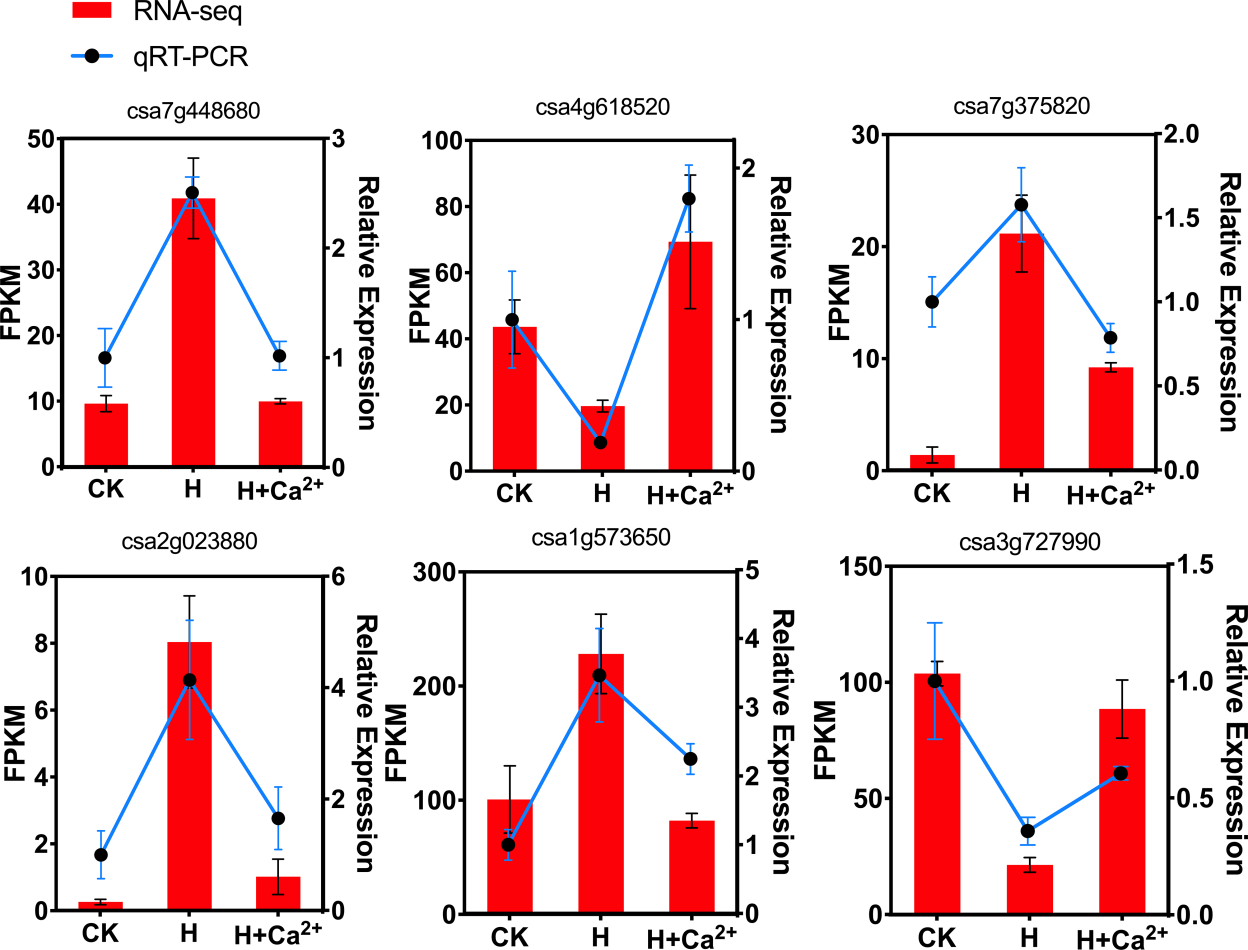
Validation of RNA-seq data by qRT-PCR. The y-axis on the left indicates the differential expression of the gene for RNA-seq, and the y-axis on the right indicates the differential expression of the gene for qRT-PCR. cDNA analysis was performed by quantitative reverse transcription-polymerase chain reaction (qRT-PCR) amplification with specific primers designed by PRIMER 3 (**Supplementary Table S8**).

To vertify the RNA-seq data, 6 DEGs related to auxin-responsive protein family (csa7g448680), ABA signaling (csa4g618520), ethylene response factors (csa7g375820), lipoxygenase 1 (csa2g023880), bZIP (csa1g573650) and WRKY (csa3g727990) were selected and quantified by qRT-PCR (**Figure 8**). Overall, the qRT-PCR data were consistent with DEGs results and indicated that RNA-seq for counting transcripts reflects transcript abundance and can be used for gene expression analysis.

## 4 Discussion

Hypoxia, including waterlogging and flooding, is a serious abiotic stress that affects plant growth and causes yield reduction (Ambros et al., 2022). Because of the weak root system and root regeneration, cucumber seedlings are particularly sensitive to hypoxic stress (Qi et al., 2019). Nowadays, the rapid development of sequencing technology provided us with more analytical tools at the molecular level to study the regulatory mechanisms of plants in response to hypoxia (Liu et al., 2021). It is well known that the expression of genes involved in plant growth, development, biotic and abiotic stress response is regulated by transcriptional and post-transcriptional levels, and the miRNAs are usually associated with post-transcriptional regulation by cleaving mRNA transcripts or inhibiting translation (Liu et al., 2018). In this study, the transcriptomes and miRNAs of cucumber cultivar “Jinchun No.2” were analyzed to investigate the mediation mechanism of exogenous calcium to alleviate hypoxic stress. A list of differentially expressed genes and miRNAs related to hormone signaling, transcription factors, calcium signaling, and glycolysis were identified and comprehensively profiled.

### 4.1 The role of plants hormone signaling in the response to hypoxia and exogenous calcium

The major function of plant endogenous hormones is to regulate plant growth, development and fruit ripening, and also could improve the tolerance of the plant to environmental factors, such as hypoxic stress (Li et al., 2021). Ethylene, as a gaseous phytohormone and a signal of hypoxia, could be significantly increased by hypoxic stress in plants (Visser and Pierik, 2007) and affect the expression of genes related to ethylene synthesis, ROS-signaling, and antioxidant system, as well as promote the adventitious formation and cell wall degradation (Khan et al., 2020). On the other hand, ethylene also could improve the hypoxic tolerance of pretreated plants (Voesenek et al., 2016). In cotton, hypoxic condition stimulates the expression of *ACS* and *ACO* genes, leading to more ethylene synthesis and aerenchyma formation in hypoxia-resistant genotypes (Pan et al., 2022). Some other ethylene-related genes also have been identified under hypoxia, such as ethylene insensitive protein (*EIN3*) and ethylene receptor gene (*ERF1*) in *Phalaris arundinacea* (Wang et al., 2021b) and group VII Ethylene-Responsive Factors (ERF-VII) in *Arabidopsis thaliana* (Tang et al., 2021). Previous studies also indicated that auxin-responsive genes (IAA and SAUR) and auxin response factor genes (ARFs) participated in the process of the “auxin signaling pathway” to strengthen plant resistance to abiotic stress (Xie et al., 2020). In tomato, ethylene accumulation could induce auxin transport and accumulation in the stem, which results in the growth of new root system under flooding (Vidoz et al., 2010). Moreover, similar discoveries also have been reported in cucumber. For example, the ethylene accumulation and auxin biosynthesis in cucumber, which is mediated by upregulating *CsACS1*, *CsACS2*, and *CsACO5*, could promote adventitious roots (ARs) formation and enhance hypoxic tolerance under waterlogged conditions (Qi et al., 2019). These observations indicate that ethylene is often involved in the response to hypoxic stress in cucumbers along with auxin. A large number of DEGs related to ethylene and auxin pathway were observed in the current study. Under hypoxia, most DEGs involved in the ethylene signaling pathway were upregulated. Although the expression of some *ACO* and *ERFs* genes were downregulated, we also found four ERFs genes (csa2g177210, csa3g017320, csa3g135120, and csa4g641590) and some other genes were stimulated by exogenous calcium treatment (**Figure 4** and **Supplementary table S3**). As for the auxin signaling pathway in our study, two auxin-responsive genes (SAUR-like genes) were significantly upregulated under hypoxia but five auxin-responsive genes were downregulated under hypoxia + CaCl_2_ treatment. Taken together, hypoxic stress could increase the expression of ethylene and auxin-related genes, and exogenous calcium could mitigate the hypoxic stress injury to cucumber seedlings by regulating the expression of these related genes (**Figure 4** and **Supplementary table S3**).

Other plant hormones signaling pathways, such as ABA (abscisic acid signaling), GA (gibberellin signaling), SA (salicylic signaling), BA (6-benzylaminopurine signaling), cytokinin, and JA (jasmonate signaling pathway), were also significantly enriched by analyzing our transcriptome data using Mapman software. Under waterlogging or flooding, the first two stress signals involve ethylene and ROS, and then the hormonal network including ABA and GA. In the modulation of elongation growth under flooding stress, ethylene, ABA and GA usually act as a trigger, repressor, and promoter, respectively (Voesenek and Bailey-Serres, 2015). More decrease in ABA content was observed in rapidly elongating *R. palustris* under submergence stress, compared to that slowly elongating ecotypes (Chen et al., 2010). Three phytohormones content (ABA, IAA, and SA) in roots of S. *tonkinensis* seedlings showed a downward trend from 0 to 72 h in response to waterlogging stress (Chen et al., 2022). In more flooding tolerant *Arabidopsis*, the genes associated with ABA and ROS were found to have more activity, such as *ABA REPRESSOR 1* (*ABR1*) (Voesenek et al., 2016). The ABA-induced ROS signaling also could activate Ca^2+^-permeable channels and simulate Ca^2+^ across the plasma membrane from internal stores (Danquah et al., 2014). We also have found many DEGs related to ABA signaling pathway were significantly upregulated by exogenous calcium in this study, including *HYR1* (csa4g618520) and *ABRs* (csa5g352640, csa5g578990, and csa5g579000) (**Figure 4** and **Supplementary table S3**). In the previous research of rice, the upregulation of the expression of mRNA encoding *GA20ox* was associated with increased concentrations of bioactive *GA1* and *GA4* in internode tissues (Ayano et al., 2014). A transcriptome study of soybean revealed that ABA signaling-related genes were upregulated, while GA signaling-related genes were downregulated under flooding stress. In addition, exogenous GA treatment can partially alleviate the germination of soybean seeds inhibited by flooding or hypoxia (Zhou et al., 2021). In our experiments, a majority of DEGs involved GA signaling pathway were also downregulated under hypoxic stress and exogenous calcium treatment (**Figure 4** and **Supplementary table S3**). These results are consistent with previous studies and suggest that exogenous calcium could increase the hypoxic tolerance of cucumber seedlings through ABA and GA signaling pathways.

Previous research revealed that cytokinin can regulate the formation of parthenocarpic cucumber fruit (Wang et al., 2021a) and jasmonic acid can improve the resistance of cucumber to aphid infestation (Qi et al., 2020). Moreover, the upregulated jasmonic acid metabolism-related genes *(LOX8*, *AOS1*, *AOC1*, and *JAR1*) and downregulated cytokinin metabolism-related genes (*IPT5-2*, *LOG1*, *CKX5*, and *ZOG2*) are involved in the regulation of adventitious roots growth in wheat under waterlogged conditions (Nguyen et al., 2018). In our transcriptome data, hypoxic stress upregulated the expression of genes related to the jasmonates pathway, such as *LOX1* (csa2g023880, and csa2g023890) and *JAZ5* (csa3g645940), but one cytokinin synthase related gene (IPT5, csa6g237640) was downregulated. However, most of jasmonates and cytokinin pathway-related genes were downregulated under hypoxia with exogenous calcium treatment (**Figure 4** and **Supplementary table S3**).

### 4.2 The role of transcription factors, calcium signaling, and glycolysis pathway in the response to hypoxia and exogenous calcium

The expression changs of transcription factors (TFs) play key roles in gene regulation and the abiotic response in plants (Licausi et al., 2011). It had been reported that the transcription factor WRKY, MYB, NAC, and bZIP partially involved in heat stress responses (Qi et al., 2021). We also had identified a large number of TFs by transcriptome data, and these differentially expressed transcription factors might affect the hypoxic resistance in cucumber.

The overexpression of *spMYB* in transgenic tobacco plants significantly elevated tolerance to salt and drought stress (Li et al., 2014). Liao et al. (2017) discovered that the MYB30 transcription factors, as a regulator of cytosolic free Ca^2+^, could positively regulate the oxidative stress response in *Arabidopsis*. In our experiment, we also discovered an MYB30 gene (csa1g009700) was significantly expressed under hypoxic stress, which suggested that MYB30 may regulate multi-stress responses in a variety of plants, including hypoxic stress and cucumber. Applying exogenous calcium, some MYB TFs, including MYB116 (csa1g042350), MYB84 (csa1g575180), and MYB4R1 (csa3g113280), were observed upregulated (**Figure 5** and **Supplementary table S4**). We postulate that these MYB TFs might involve in the positive regulation of exogenous calcium in response to hypoxic stress in cucumbers. WRKY TFs families are considered to be the largest and most important family of transcription factors in plants and associate with the tolerance of various stresses, such as pests, pathogens, and hypoxia (Hsu et al., 2013). Recently studies have shown that hypoxic stress could induce the expression the WRKY12, WRKY22 and WRKY33 in *Arabidopsis thaliana* (Tang et al., 2021). As expected, the expression of WRKY48 (csa3g168410) and WRKY23 (csa7g407820) were found to be increased significantly under hypoxia, and exogenous calcium increased the expression of WRKY49 (csa4g296160), WRKY70 (csa3g727990), WRKY71 (csa6g139770) and WRKY75 (csa4g012390) (**Figure 5** and **Supplementary table S4**).

Calcium signaling is very important for the regulation of stress response in plants, and the calcium influx is usually triggered by ROS signaling in response to salt, heat, drought and hypoxia (He et al., 2015; Liao et al., 2017; Ma et al., 2021). There are roughly three calcium sensors in plants to sense and transduce calcium signaling, namely calmodulin (CaM) and calmodulin-like proteins (CMLs), Ca^2+^-dependent protein kinases (CDPKs/CPKs), and calmodulin-dependent protein kinases (CaMKs) (Tortosa et al., 2019). Previous research showed that LlCaM3 could involve in heat resistance in lily (*Lilium longiflorum*) by interacting with LlWRKY39 (Ding et al., 2021). The osmoprotectant effect is another important function of calcium and had been demonstrated for a long time (Gerard, 1971). In *Arabidopsis thaliana*, AtCPK1 and AtCPK6 overexpression improved salt and drought tolerance, while AtCPK21 and AtCPK23 were related to the osmotic stress tolerance (Yip Delormel and Boudsocq, 2019). In this study, we identified many calcium signaling genes were upregulated under hypoxia + CaCl_2_ treatment, such as CaM1 (csa2g011480), CPK21 (csa6g513780), and CPK-related kinase 1(csa4g430830), compared to hypoxia treatment (**Figure 6** and **Supplementary table S5**). The glycolysis metabolic pathway is the main way for plants to produce energy under anoxic conditions. The expression of glycolysis-related genes, including pyruvate decarboxylase (PDC), alcohol dehydrogenase (ADH), and alanine aminotransferase (AlaAT), would induce by hypoxic stress (Rocha et al., 2010; Licausi et al., 2011). The genes encoded aldolase (csa3g750920), phosphoglycerate/bisphosphoglycerate mutase (csa6g151120), phosphoenolpyruvate carboxylase (csa4g627210), and pyruvate kinase (csa5g580610) were upregulated under anoxic stress and were further upregulated after applying exogenous calcium in our study. These findings suggested exogenous calcium could alleviating the injury to cucumber caused by hypoxic stress *via* the calcium signaling pathway and enhancing the expression of those glycolysis-related genes.

### 4.3 The role of miRNAs in the response to hypoxia and exogenous calcium

Some essential and functionally important regulatory factors such as miRNAs, are most studies class of small RNA, and miRNAs can interact with their target genes to regulate plant growth, development, and stress responses (Wu et al., 2015). It has been reported some miRNAs, such as miR164d, miR167e, miR171f, and miR172c, are involved in the response to *C. cassiicola* in cucumber (Wang et al., 2018b). In the experiment of cucumber against powdery mildew, 156 known and 147 novel miRNAs were identified, including miR172c-3p, miR395a-3p, miR395d-3p, and miR398b-3p (Xu et al., 2020). When exogenous spermidine was used to mitigate heat stress in cucumber, 107 known and 79 novel miRNAs were observed to be differently expressed, such as miR2275-5p, miR394a, miR479b, and miR6475 (Wang et al., 2018c). In the analysis of the small RNA transcriptome of maize roots under submergence stress, 45 differentially expressed miRNAs were found, and these miRNAs were related to hormone signaling, redox homeostasis, miRNA biogenesis, and protein transport (Azahar et al., 2020). In our study, 56 known miRNAs and 30 novel miRNAs were identified, and those hypoxia-responsive miRNA families mentioned above were also found to be differently expressed in our dataset (**Figure 7**). For instance, the downregulation of miRNA164c-3p was consistent with a previous report about maize seedlings (Azahar et al., 2020). The report showed that some miRNAs belonging to miR164 family, such as miR164a, miR164b-5p and miR164c-5p, were down-regulated under submergence stressed. It is well known that the expression patterns of miRNAs do not always coincide with the expression patterns of target genes, so further experiment is needed to validate the functions of related genes.

## 5 Conclusion

Our transcriptome study highlights the effect of DEGs, miRNAs and their putative target genes associated with exogenous calcium to alleviate hypoxic stress in cucumber seedlings. The functional and signaling pathway analysis of DEGs revealed that exogenous calcium could associate with the hormone signaling pathway (ethylene, ABA, IAA, and cytokinin), transcription factors (MYB, MYB-related, bHLH, bZIP and C2C2-CO-like), calcium signaling and glycolysis pathway to mitigating hypoxic stress in cucumber seedlings. In addition, we also identified 56 known miRNAs, 30 novel miRNAs and their predicted target genes that responded to hypoxia and exogenous calcium. In summary, this work combined with the transcriptome and miRNA data will help and guide us to better understand and investigate the resistance mechanism by which exogenous calcium alleviates hypoxic stress in cucumber seedlings.

## Data availability statement

The datasets presented in this study can be found in online repositories. The names of the repository/repositories and accession number(s) can be found below: National Center for Biotechnology Information (NCBI) BioProject database under accession number PRJNA910791.

## Author contributions

JYu: Project administration, funding acquisition and methodology. QZ: Conceptualization and experiments designing. LH: Experiment execution, writing, and editing the draft. JYan: Statistical analysis, writing, and editing the draft. XD: participated in the experiment and review the draft. HJ: reviewing and editing the draft. HZ: Reviewing and editing the draft. JC: Statistical analysis. All authors contributed to the article and approved the submitted version.
